# Microfluidic Microwave Sensor Based on a Twisted Cross-Shaped Structure for Glucose Detection

**DOI:** 10.3390/s25133974

**Published:** 2025-06-26

**Authors:** Yun Ma, Chenxi Zhao, Chaojun Chen, Quanlin Guo, Junxi Zhu, Dezhi Gou, Chuan Liu

**Affiliations:** School of Electronic Information Engineering, China West Normal University, Nanchong 637009, China; my_cwnu@163.com (Y.M.); chenxizhaoxt@163.com (C.Z.); chinachenchaojun@163.com (C.C.); cwnu_gql@163.com (Q.G.); cwnujunxiz@163.com (J.Z.); gdz_scu@163.com (D.G.)

**Keywords:** microwave sensor, glucose detection, microfluidics

## Abstract

Microwave sensors have shown significant potential for glucose detection. However, temperature fluctuations have the drawback of interfering with the measurement accuracy of microwave sensors. In this work, a novel microwave sensor based on the twisted cross-shaped structure for glucose detection is designed, applying a microfluidic device for precise temperature control. The operating frequency of this sensor is 6900 MHz, and its sensitivity is 0.54%. The experimental results show that under the constant temperature, due to an increase of 0.2 mol/L in glucose concentration, the resonance point frequency of the scattering parameter (S_21_) is shifted approximately 10 to 60 MHz. With the increase in glucose concentration, the resonance amplitude of the S_21_ increases, while the frequency shift decreases. At 9 °C, when the glucose concentration is within the range of 0.05 to 0.2 mol/L, the frequency shift is about 50 MHz. Under the constant glucose concentration, due to an increase of 10 °C in temperature, the resonance point frequency of the scattering parameter (S_21_) is shifted approximately 10 to 130 MHz. At the 0.01 mol/L glucose concentration, when the temperature is within the range of 9 to 15 °C, the frequency shift is about 130 MHz. This work provides a novel approach for glucose monitoring and also offers experimental support for the application of microwave sensors in biomedicine.

## 1. Introduction

Microwave sensors have been widely used to measure the dielectric constant of various liquids due to their high sensitivity and non-contact measurement capability [1,2]. These microwave sensors are typically based on frequency-locked loops (FLL), frequency-locked rings, and multiple complementary split-ring resonators (CSRRs) [3,4]. The low-cost CSRR-based sensor stands out for its affordability and reliable measurement of the dielectric constant of liquid samples [5]. Furthermore, highly sensitive microwave sensors based on Open Complementary Split Ring Resonators (OCSRRs) have been proposed for measuring the complex permittivity of liquid solutions, offering high sensitivity and precision [6]. These advancements highlight the significant potential and ongoing innovation in microwave sensing for liquid analysis. With the development of microwave sensor technology, their application has extended to glucose concentration detection [7].

The dielectric constant of glucose solutions varies with concentration [8], allowing microwave sensors to measure glucose concentration accurately. Researchers have developed various microwave sensor-based glucose detection systems [9,10,11], some of which utilize the frequency shift in microwave resonators to reflect changes in glucose concentration and measure the dielectric constant [12]. While frequency shift sensors are sensitive, they may struggle in complex dielectric conditions compared to insertion loss sensors [13], which offer more direct measurements by detecting dielectric changes through electromagnetic wave energy loss. However, insertion loss sensors are highly sensitive to external environmental changes, which affect their stability in certain applications. Q-factor-based sensors enable high-resolution detection by monitoring variations in the quality factor of the resonator, but their performance is affected by temperature and environmental fluctuations [14]. Phase-change sensors detect substances by monitoring electromagnetic wave phase shifts, making them suitable for high-sensitivity detection in complex media, though external noise can impact stability [15]. Planar microwave sensors that utilize coupling between resonators have demonstrated superior sensitivity; however, their implementation generally demands more intricate design and fabrication processes [16].

In addition, some studies have combined microwave sensors with surface plasmon resonance technology to enhance microwave signal response and improve glucose detection sensitivity [17,18]. Some studies have combined microfluidic technology with optical methods to achieve high-sensitivity detection, but their fabrication and calibration processes are relatively complex, resulting in higher costs [19]. Other systems have integrated microwave sensors with circuit technology to develop portable, low-cost glucose monitoring devices [20,21,22]. These systems demonstrate great potential for biomedical diagnostics and monitoring. To further improve the sensitivity and accuracy of glucose detection, combining microwave sensors with microfluidic technology has become an important research direction [23,24]. Microfluidic technology enables precise control and processing of liquid samples at microscale, significantly improving sensor measurement efficiency and accuracy [25,26]. By integrating microwave sensors into microfluidic systems, researchers have achieved efficient, rapid, and low-sample-consumption glucose detection [27,28].

Although the impact of temperature on glucose concentration measurements using microwave sensors has been investigated, and several correction methods have been proposed, these approaches exhibit notable limitations in specific application scenarios. For example, the temperature compensation technique developed by Ali A. Abduljabar has a limited applicability range [29], the correction method for sodium lactate solutions proposed by Odette S. Bakam Nguenouho shows relatively large errors [30], and the method introduced by Chorom Jang for glucose solutions is constrained by a narrow measurement range [31]. Most existing studies focus on ideal laboratory conditions and do not fully account for complex temperature variations and external disturbances in practical applications. With the emergence of new microwave sensor designs, existing correction methods may no longer be fully applicable. Thus, further research is needed to develop more robust and widely applicable temperature correction strategies. In this paper, a temperature correction method tailored for microwave sensors in realistic experimental settings is investigated and presented, offering insights into complex biomedical sensing environments.

Glucose molecules are carbohydrates commonly found in biological systems and food sources [32], and are essential in human physiology and the food industry. In biomedicine, glucose serves as the primary energy source for vital bodily functions [33,34], and plays a critical role in disease diagnosis and therapy. Rapid and accurate glucose detection has become a crucial task in clinical practice [35]. Non-invasive glucose detection technologies have attracted considerable attention due to their potential to revolutionize disease management [36,37]. Among these, microwave sensors are promising due to their ability to perform mediator-free glucose detection [38], which enhances usability and minimizes patient discomfort. The physical properties of glucose solutions can be characterized by microwave scattering parameters, as they induce resonance offsets in sensor cells [39]. These studies leverage the dielectric properties of glucose solutions to calibrate and validate sensor performance, ensuring accuracy in applications such as medical diagnostics and food quality control [40].

This paper investigates the effect of temperature on glucose concentration measurements using microfluidic microwave sensors and presents a novel design with experimental validation. Unlike conventional invasive methods, the proposed sensor employs microfluidic technology to directly sample glucose from prepared solutions for glucose sensing [41,42,43]. Liquid samples are introduced into the microfluidic channels of the sensor, resulting in a resonance frequency shift that enables glucose detection. This approach offers a promising pathway for accurate and real-time glucose monitoring. By regulating temperature using a water bath system, the sensor ensures consistent and reliable measurement outcomes. This temperature-controlled environment enhances measurement accuracy and minimizes the impact of temperature fluctuations on sensing performance [44]. To evaluate the effectiveness of the proposed sensor, a series of experiments was conducted to assess the influence of temperature on microfluidic glucose assays. Glucose solutions with varying concentrations were prepared and analyzed using the sensor system. The experimental results provided valuable insights into sensor performance under different temperature conditions, demonstrating its potential for real-world glucose monitoring. The development of this microfluidic microwave sensor represents a significant advancement in glucose detection technology [45,46].

## 2. Microwave Sensor Design

### 2.1. Structure Design of Microwave Sensor Based on the Twisted Cross-Shaped Structure

**Figure 1:**
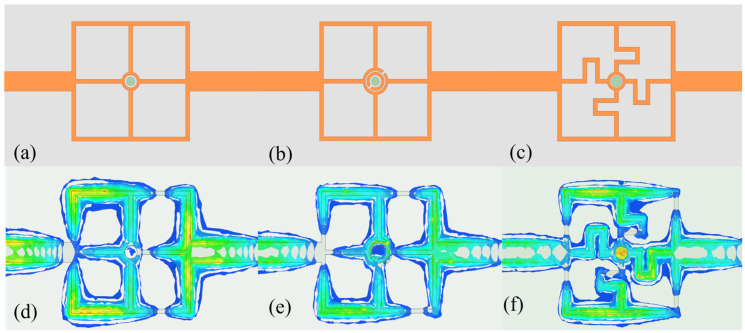
Design of different microwave sensors. (**a**) Cross-shaped model. (**b**) Cross-CSRR model. (**c**) Twisted-cross model. (**d**) Electric fields of Cross-shaped model. (**e**) Electric fields of Cross-CSRR model. (**f**) Electric fields of twisted-cross model.

To achieve optimal sensing performance, several resonator geometries were initially analyzed through full-wave electromagnetic simulations, as illustrated in Figure 1. The candidate structures included the conventional split-ring resonator (SRR) and the standard cross-shaped resonator. A systematic comparison was conducted under identical excitation conditions, focusing on electric field confinement and sensitivity to variations in the dielectric constant, as summarized in Table 1. The simulation results demonstrated that the proposed twisted cross-shaped structure exhibited the strongest localized electric field within the microfluidic channel region, thereby significantly enhancing the interaction between the electromagnetic field and the analyte. This confirmed the superior performance of the design for microwave sensing applications.

**Figure 2:**
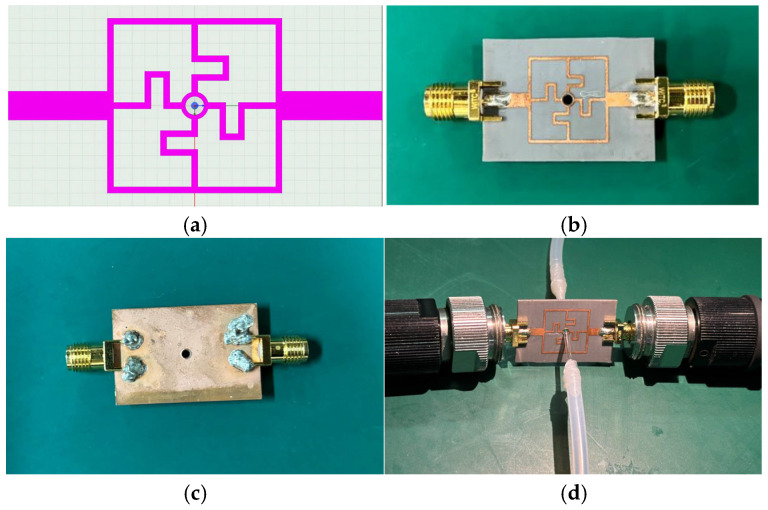
Design of microwave sensor. (**a**) Simulation model. (**b**) Top view of material object. (**c**) Bottom view of material object. (**d**) Microwave sensor channel.

This section proposes a novel microwave sensor based on the twisted cross-shaped structure. The sensor comprises three layers: the top layer is a microstrip wire structure, the middle layer is F4B with a dielectric constant of 2.2, known for its thermal stability, chemical resistance, excellent electrical performance, and low cost, and the bottom layer is copper for grounding. The sensor measures 30 mm × 20 mm with a thickness of 0.762 mm. The proposed sensor employs a bent microstrip resonator structure, which is intended to enhance the localization of the electromagnetic field in the sensing region and facilitate the generation of a distinct resonance response. Full-wave simulations conducted over the 0–10 GHz frequency range indicate a single pronounced resonance at approximately 6.9 GHz, with well-defined resonance characteristics. This frequency was therefore identified as the operational point of the structure. Field distribution analysis further reveals that the electromagnetic energy is concentrated in the vicinity of the microfluidic channel at this frequency, which is favorable for detecting subtle variations in the dielectric properties of the sample under test. Figure 2 shows a simulation model created with the High Frequency Structure Simulator (HFSS) to verify the electromagnetic characteristics of the sensor. After fabrication, SMA connectors are soldered to two PCB ports for testing with a vector network analyzer (VNA). Figure 2b,c show the front and back images of the sensor after machining. The design process prioritized performance, cost-effectiveness, and feasibility.

**Figure 3:**
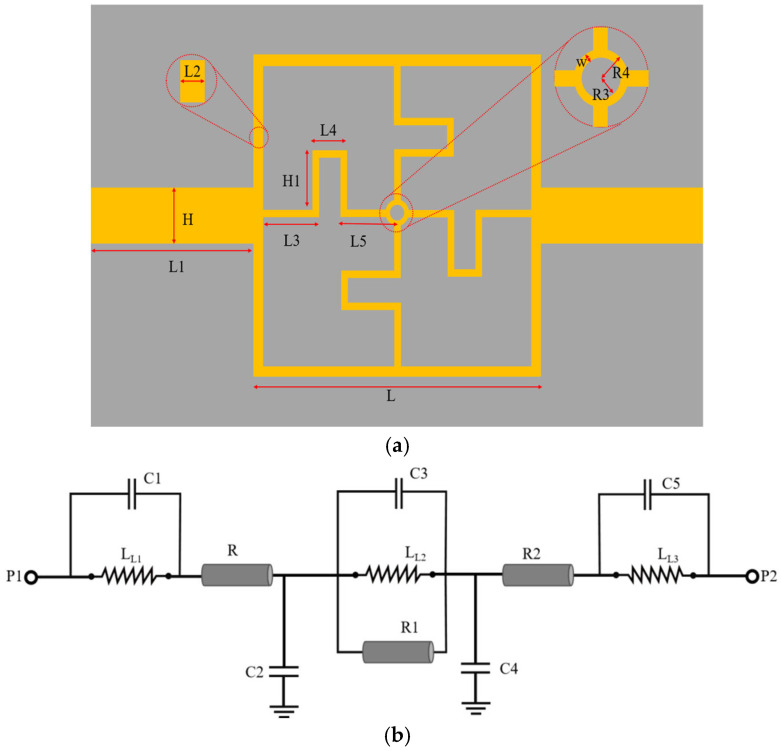
Microwave sensor structure. (**a**) Model structure. (**b**) Equivalent circuit.

Figure 3 shows the schematic and equivalent circuit of the sensor. The geometrical dimensions of the sensor are listed in Table 2, while Table 3 provides the detailed component values of the equivalent circuit, including capacitances, inductances, and resistances. A thorough analysis of the schematic and equivalent circuit of the sensor enables a more intuitive understanding of its operational principles, internal structure, and the interactions between individual components. The equivalent circuit simplifies the complex electromagnetic behavior of the sensor into a network of capacitive, inductive, and resistive elements, each representing a specific physical phenomenon. For instance, capacitance corresponds to the electric field energy storage of the sensor, inductance reflects magnetic field effects, and resistance accounts for energy dissipation. This model not only provides a theoretical foundation for understanding the operation of the sensor but also serves as a crucial tool for guiding its design optimization and performance enhancement.

Perturbation theory is used to analyze and predict circuit performance changes in the design and optimization of microwave sensors [47,48]. Here the characteristic impedance *Z*_0_ of the whole equivalent circuit can be expressed as

(1) $$\begin{array}{l} Z_{0}=\frac{1}{\frac{1}{jwL_{L1}}+\mathrm{jw}C_{1}}+R+\frac{1}{jwC_{2}}+\\ \frac{1}{\frac{1}{jwL_{L2}}+\mathrm{jw}C_{3}+\frac{1}{R_{1}}}+\frac{1}{jwC_{4}}+R_{2}\\ +\frac{1}{\frac{1}{jwL_{L3}}+\mathrm{jw}C_{5}} \end{array}$$

The formula shows that the resonance frequency *f* is related to inductance and capacitance, and *f* is calculated by the definition of resonance. Perturbation theory can be used to analyze the change in resonance frequency when *L* or *C* changes slightly [49,50]. Specifically, when the inductor *L* or capacitor *C* is perturbed, the change in resonance frequency *f* can be approximated by Taylor expansion:

(2) $$\delta f\approx (\frac{\partial f}{\partial L})\delta L+(\frac{\partial f}{\partial C})\delta C$$

**Figure 4:**
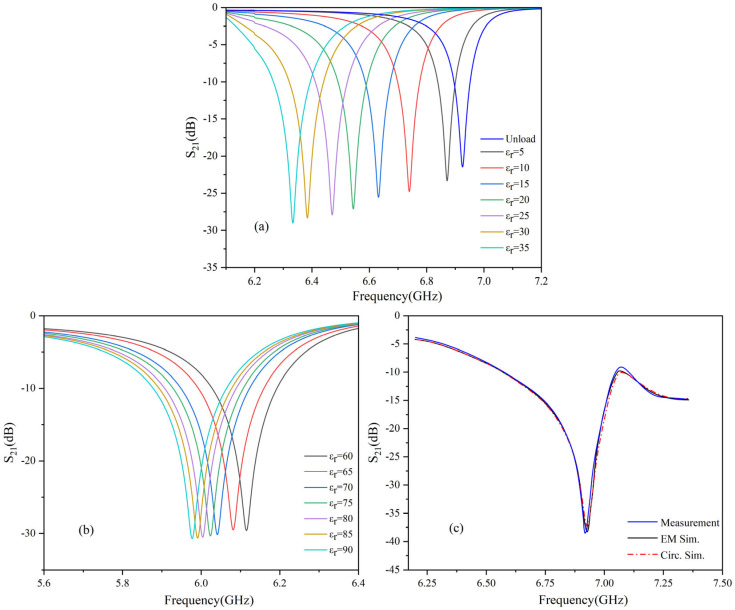
*S*_21_ parametric simulation and testing. (**a**) Low−dielectric HFSS simulation. (**b**) High−dielectric HFSS simulation. (**c**) Measurement and simulation of transmission response.

Figure 4a,b present the HFSS simulation results of the *S*_21_ parameter for various liquid dielectrics with different permittivities. In the simulation, the dielectric constant was incrementally varied in steps of 5, while the loss tangent of the F4B substrate was fixed at 0.0012. The results indicate that, in the absence of a dielectric load, the resonance frequency *f* is 6.9 GHz. As the permittivity of the liquid medium changes, a corresponding shift in the resonance frequency is observed. Specifically, when the permittivity is set to 5, the resonance frequency decreases to 6.87 GHz. For each increment of 5 in the permittivity, the frequency shift is approximately 0.1 GHz. Within the current range of permittivity values tested, the relationship between the dielectric constant and resonance frequency exhibits a regular pattern. However, as the dielectric constant increases further, the frequency shift decreases, resulting in reduced sensor sensitivity.

Figure 4c compares the *S*_21_ parameters obtained from the equivalent circuit model, HFSS simulations, and experimental measurements using a vector network analyzer (ZNB 40, Rohde & Schwarz, Munich, Germany) after fabrication. To validate the accuracy of the proposed equivalent circuit model, ADS simulation software (Advanced Design System 2020) was utilized. As indicated by the dashed line in Figure 4c, the simulated resonance frequency of the unloaded equivalent circuit is 6.9 GHz, which matches the simulated resonance frequency of the microwave sensor. Both simulation curves exhibit the same resonance frequency in the *S*_21_ parameter. The ADS and HFSS simulation results align closely with the experimental data, validating the accuracy and reliability of the equivalent circuit model in predicting the performance of the sensor.

For the purpose of comparing sensors with each other, the responsiveness of the resonance frequency *f* to changes in the dielectric constant Δ*ϵ* is described using the frequency sensitivity *S*, which is defined as follows for the measurement of the dielectric constant [51].

(3) $$S=\frac{\Delta f}{\Delta \epsilon \cdot f_{0}}\times 100\%$$

where Δ*f* is the change in frequency, Δ*ϵ* is the change in dielectric constant, and *f*_0_ is the initial resonance frequency. By calculating the frequency sensitivity, one can assess how sensitive the RF circuit is to changes in the dielectric constant.

The quality factor *Q_MUT_* is an important parameter that measures the performance of a resonator, defined as the ratio of stored energy to dissipated energy [52,53]. For the resonance circuit of this sensor, the quality factor *Q_MUT_* is related to the unloaded quality factor *Q_U_* involving *S*_21_ by

(4) $$Q_{MUT}^{-1}=\frac{1}{Q_{U}[1-10^{\frac{S_{21}(dB)}{20}}]}$$

where *Q_U_* = *f*_r_/*f*_Δ3dB_, *f*_r_ is the resonance frequency and *f*_Δ3dB_ is the −3 dB bandwidth. A high-quality factor indicates that the resonance circuit has low energy loss and high selectivity.

Complex permittivity ε* describes the frequency response characteristics of the dielectric material, capturing its polarization behavior [54,55]. It is typically expressed as

(5) $$\varepsilon^{*}=\varepsilon_{\infty }+\frac{\Delta \varepsilon_{1}}{1+{\left(j\omega \tau_{1}\right)}^{\beta }}+\frac{\Delta \varepsilon_{2}}{1+j\omega \tau_{2}}$$

where *ε_∞_* is the high-frequency limit permittivity, Δ*ε*_1_ and Δ*ε*_2_ are the dielectric constant changes for two relaxation processes, *τ*_1_ and *τ*_2_ are the corresponding relaxation times, *β* is the relaxation distribution parameter, and *ω* is the angular frequency.

### 2.2. Electric Field Analysis

**Figure 5:**
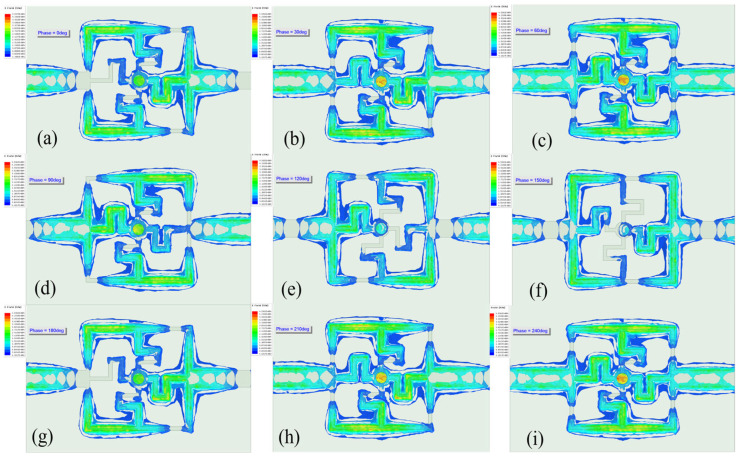
Electric fields of microwave sensors at different phases. (**a**) 0 deg. (**b**) 30 deg. (**c**) 60 deg. (**d**) 90 deg. (**e**) 120 deg. (**f**) 150 deg. (**g**) 180 deg. (**h**) 210 deg. (**i**) 240 deg.

Electromagnetic simulation software ANSYS HFSS (ANSYS 18.2) was used to simulate the electric field of a microwave sensor. As shown in Figure 5, the simulation results indicate that the microchannel is a significant concentration area for the electric field. The electric field strength in the microchannel is 1.524 × 10^5^ V/m, much higher than the 6.743 × 10^3^ V/m in other parts of the circuit. This electric field distribution ensures effective interaction between the microwave signal and glucose molecules, promoting resonance shift.

**Figure 6:**
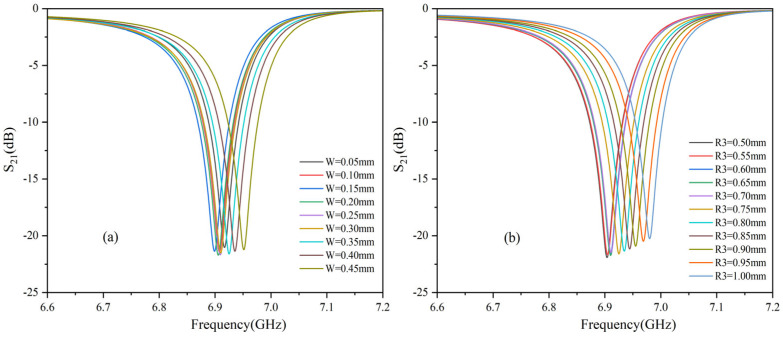
*S*_21_ Changes with different parameter values. (**a**) W from 0.05 mm to 0.45 mm. (**b**) R3 from 0.50 mm to 1.00 mm.

Simulations of the microstrip circuits show a high-intensity electric field within the microchannels, significantly stronger than in other circuit regions. Electric field maps, as shown in Figure 5, reveal that this distribution is critical for microwave sensors, directly affecting the interaction with glucose molecules. The design and optimization process used HFSS simulations to evaluate how key parameters, including microstrip line width, microchannel radius, resonator length, and spacing between the microstrip and microchannel, influence electric field strength. The microstrip width (w) was optimized to achieve the target resonance frequency and ensure efficient electromagnetic wave propagation, while the microchannel height (R3) was fine-tuned to concentrate the electric field within the channel and prevent leakage. The optimization results for the microstrip width and microchannel radius are shown in Figure 6. After multiple optimization iterations, the optimal geometric parameters were identified, resulting in a significant enhancement of electric field strength and a marked improvement in sensor sensitivity and accuracy.

The electric field distribution in the microchannel of the microwave sensor is a key factor affecting effective signal detection and glucose molecule interaction. Understanding the behavior of the electric field is important for optimizing the performance and application of microwave sensors, promoting the development and application of microwave sensor technology.

## 3. Glucose Solution Detection

### 3.1. Glucose Concentration Detection Experiment

**Figure 7:**
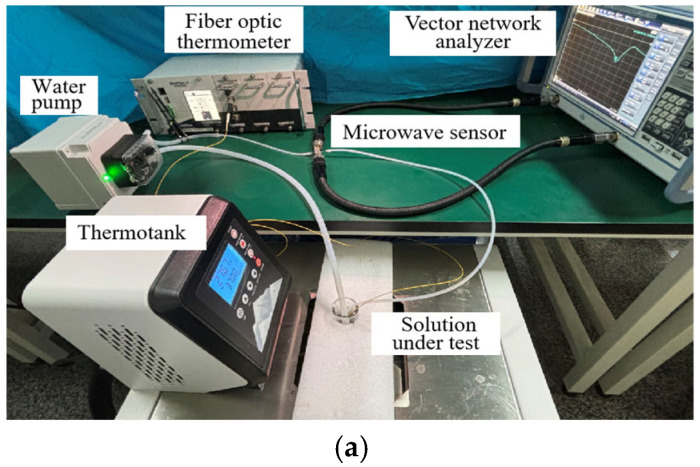

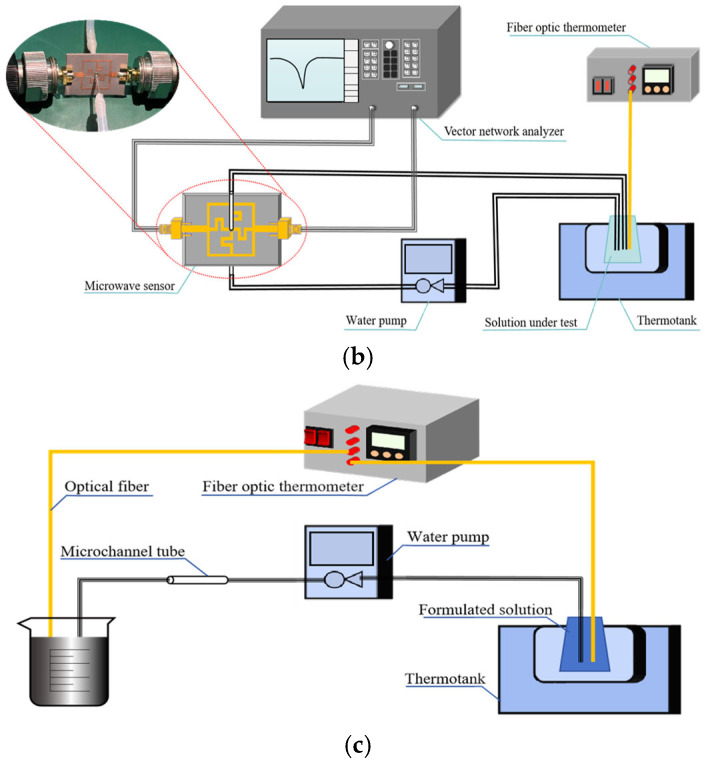
Experimental equipment for microwave sensing system. (**a**) Physical drawings of experimental equipment. (**b**) Schematic diagram of experimental equipment. (**c**) Temperature verification diagram.

This paper introduces a microfluidic microwave sensor for detecting glucose solution concentrations. The physical diagram of its experimental equipment is shown in Figure 7a. To verify the performance of the sensor, glucose solution samples with concentrations of 0.05 mol/L, 0.1 mol/L, 0.2 mol/L, 0.4 mol/L, 0.6 mol/L, 0.8 mol/L, and 1.0 mol/L were prepared in the laboratory. These solutions were prepared by precisely weighing glucose, dissolving it in a quantified amount of deionized water, and introducing the glucose solution into a microfluidic tube for testing.

To investigate the physical properties of these glucose solutions at different temperatures, temperatures were set to 9 °C, 15 °C, 25 °C, and 35 °C. During the experiment, a sufficient amount of glucose solution was added to a volumetric bottle. The bottle was then placed in a thermostatic box for a water bath to control the temperature. The temperature of the glucose solution in each experiment was measured and recorded with a fiber optic thermometer to ensure accurate data acquisition. All solutions were prepared under the same environmental conditions to ensure repeatability and accuracy. Repeated tests at different temperatures allowed observation of the effect of temperature on the properties of the glucose solution, resulting in a comprehensive understanding of its physicochemical properties under various conditions.

The schematic diagram, shown in Figure 7b, consists of a water pump, a thermostat, a fiber optic thermometer, a vector network analyzer (VNA), and a microwave sensor. The water pump moves the glucose solution from the volumetric bottle into the microflow tube for circulation. The vector network analyzer measures the transport parameter *S*_21_ of the glucose solution. After each measurement, the microfluidic tube was cleaned with deionized water to restore the unloaded resonance. All measurements were performed in microfluidic channels and under constant temperature conditions to minimize interference from temperature fluctuations or solution evaporation. This setup ensures minimal temperature variation, allowing for consistent and reliable glucose concentration measurements.

The microfluidic channel is integrated into the microwave sensor to enable controlled glucose solution flow, enhancing the sensitivity and accuracy of the detection process. Figure 7b provides a flow diagram of the system, detailing the individual components and their connections. The use of microwave sensors enables accurate measurement of changes in the electrical properties of the solution, allowing inference of glucose concentrations. This method not only achieves high sensitivity but also enables fast and real-time detection, with broad application prospects. To ensure a constant water temperature within the micro-channel, a temperature validation experiment was conducted, as shown in Figure 7c. The experimental results demonstrate that, at the same pump speed, the temperature on both sides of the microfluidic channel remains uniform.

Combining microfluidic technology with microwave sensing technology accurately detected different glucose solution concentrations. Maintaining a constant temperature and using equipment such as fiber optic thermometers and vector network analyzers ensured accurate and reliable measurements. This method provides important technical support for further research and application.

### 3.2. Experimental Results Analysis

Glucose solutions of varying concentrations were tested under constant temperature conditions using microwave sensors, with their transport parameter S21 measured by the VNA. Four temperature points were selected for experimental testing at 9 °C, 15 °C, 25 °C, and 35 °C. The experimental results for each temperature point are shown in Figure 6. By adjusting the VNA, the *S*_21_ parameters for different glucose solution concentrations were accurately measured and recorded.

**Figure 8:**
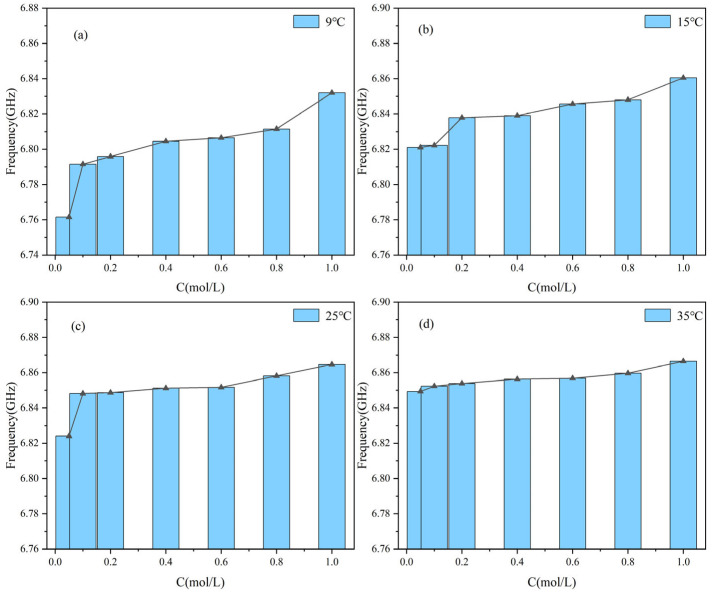
The resonance frequency of glucose solution changes with different concentrations at constant temperature: (**a**) 9 °C; (**b**) 15 °C; (**c**) 25 °C; (**d**) 35 °C.

Figure 8 illustrates the resonant frequency response of the proposed sensor to varying glucose concentrations under different temperature conditions. The experimental results demonstrate that as the glucose concentration increases from 0.05 mol/L to 1.0 mol/L, the resonant frequency exhibits an overall upward trend. At 9 °C and 15 °C, the frequency shift is more pronounced, with stronger linearity, indicating higher sensitivity to concentration changes. In contrast, at 25 °C and 35 °C, the frequency variation tends to saturate, and the sensitivity is significantly reduced. These findings indicate that the sensor exhibits a degree of temperature dependence during concentration detection, highlighting the necessity of incorporating temperature compensation strategies in practical applications to ensure measurement accuracy and stability.

**Figure 9:**
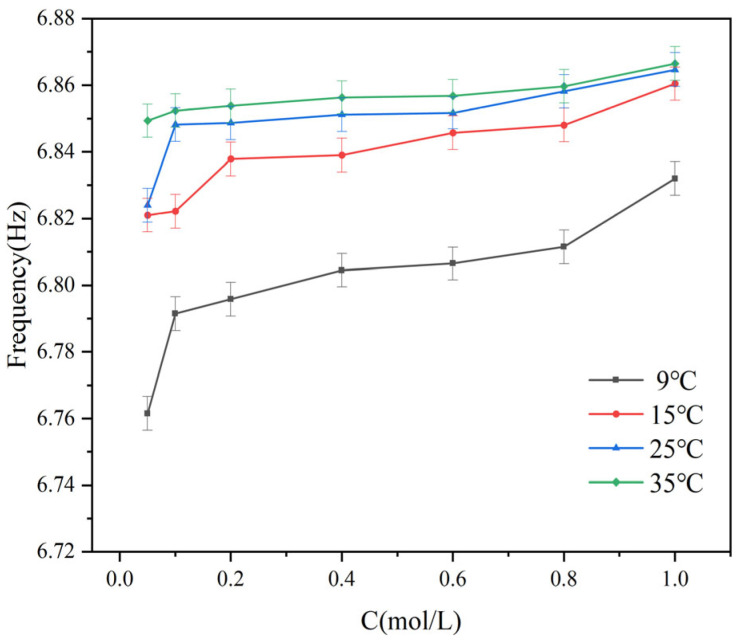
Variation in the frequency point of the *S*_21_ parameter of glucose solutions with concentration at the same temperature.

Figure 9 presents the variation in the resonance frequency of the sensor under different glucose concentrations and four ambient temperature conditions. Experimental results show that at all tested temperatures, the resonance frequency increases nonlinearly as the glucose concentration rises from 0.05 mol/L to 1.0 mol/L. In the low concentration range, specifically from 0.05 mol/L to 0.2 mol/L, the frequency increases rapidly, indicating higher detection sensitivity. As the concentration continues to increase, the rate of frequency change gradually decreases, and the response curve becomes flatter. This saturation behavior is particularly evident at 25 °C and 35 °C. These results further confirm that the resonance frequency response of the sensor exhibits significant dependence on both ambient temperature and solution concentration.

The results of Figure 8 and Figure 9 show that under the same temperature conditions, temperature and concentration jointly affect the frequency response and transmission characteristics of the system. At high temperatures, the *S*_21_ values are relatively stable, and the system response changes little. This indicates that system performance is less affected by concentration changes at higher temperatures. Under low temperature conditions, the *S*_21_ values change significantly, indicating that concentration changes have a greater impact on system performance. To express this relationship more intuitively, a function is used to represent the relationship between *S*_21_ and concentration, fitting the experimental data. The correlation is as follows:

(6) $$F_{21}(c)=A_{1}\cdot c^{3}-B_{1}\cdot c^{2}+C_{1}\cdot c+D_{1}$$

Among them, *A*_1_, *B*_1_, and *C*_1_ are empirical parameters, with their specific coefficient values shown in Table 4, *F*_21_(*c*) is the value of the resonance point of *S*_21_, and *c* is the glucose concentration. This functional relationship clearly reflects the variation in the resonance point of *S*_21_ at different concentrations.

**Figure 10:**
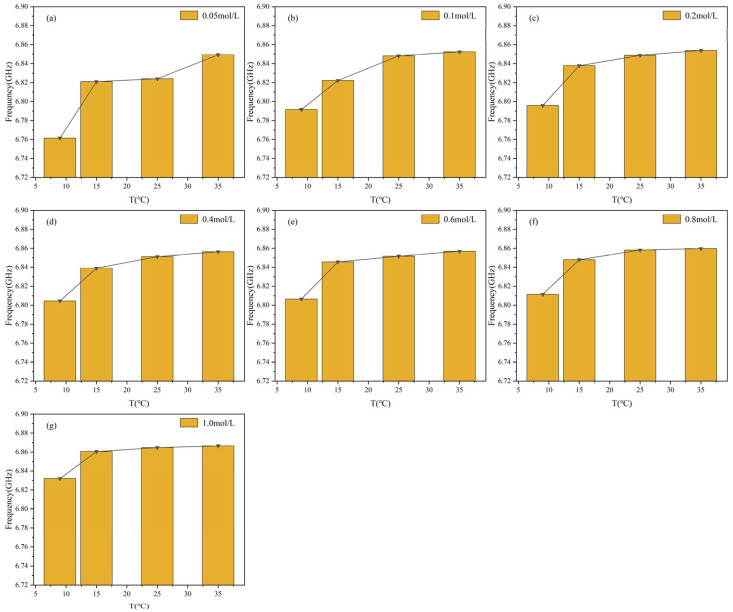
The resonance frequency of glucose solution changes with different temperatures at constant concentrations: (**a**) 0.05 mol/L; (**b**) 0.1 mol/L; (**c**) 0.2 mol/L; (**d**) 0.4 mol/L; (**e**) 0.6 mol/L; (**f**) 0.8 mol/L; (**g**) 1.0 mol/L.

To explore how temperature affects microwave sensor measurements, the temperature of glucose solutions was varied while maintaining the same concentration, and their frequency response parameters were measured and recorded. Figure 10 illustrates the effects of four different temperatures on the resonance frequency parameter at various concentrations. The experimental results showed that, at constant glucose concentrations, the resonance frequency of the sensor increased monotonically with rising temperature. As illustrated in Figure 10a, when the temperature increased from 9 °C to 35 °C, the resonance frequency shifted from approximately 6.76 GHz to 6.85 GHz. A similar behavior was observed in Figure 10g, where the frequency increased from around 6.83 GHz to 6.89 GHz. It was noted that the frequency shift was more significant in the range of 9 °C to 15 °C, whereas a reduced rate of change was observed between 15 °C and 25 °C. These results demonstrate the temperature sensitivity of the sensor’s resonance response, indicating that temperature-induced frequency drift must be compensated in practical glucose sensing applications to maintain measurement accuracy and reliability.

**Figure 11:**
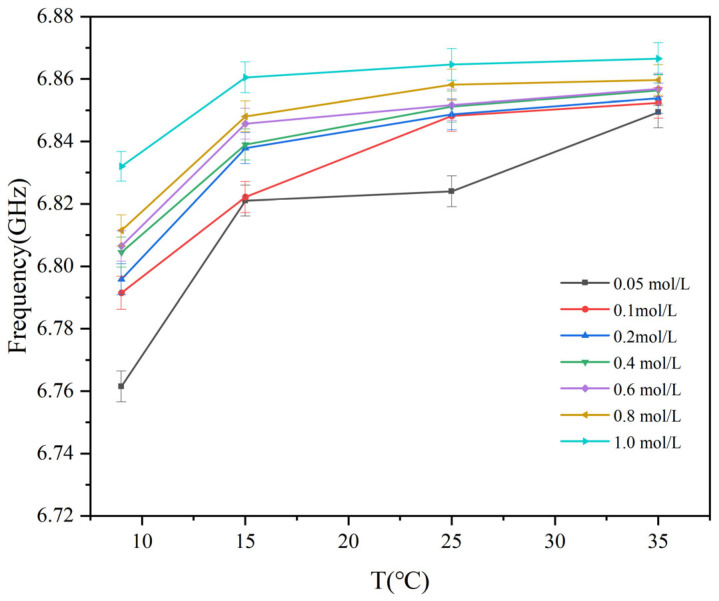
Variation in the frequency point of the S_21_ parameter of glucose solutions with temperature at a constant concentration.

Figure 11 illustrates the frequency response trend with temperature at various glucose concentrations. The resonance frequency increases with rising temperature, stabilizing in the range of 25 °C to 35 °C. When the temperature increased from 9 °C to 15 °C, the resonance frequency of the glucose solution exhibited a rapid rise. As the temperature exceeded 25 °C, the frequency tended to gradually stabilize. Between 25 °C and 35 °C, the frequency continued to increase at a slower rate and eventually stabilized at approximately 6.86 GHz at 35 °C. These results indicate that the sensor’s sensitivity to temperature variations decreases with increasing temperature, resulting in a more stable frequency response and enhanced thermal stability.

Figure 10 and Figure 11 emphasize the impact of temperature on frequency variation and system stability, particularly noticeable at lower temperatures. Lower concentrations exhibit significant variability in *S*_21_ values, indicating notable differences across different temperature profiles. Higher concentrations lead to tightly converging curves across all temperature variations, emphasizing improved consistency in transmission characteristics. This indicates reduced sensitivity to temperature fluctuations and enhanced stability at higher concentrations. To express this relationship more intuitively, a function is used to model the correlation between *S*_21_ and temperature, fitting the experimental data. The correlation is as follows:

(7) $$F_{21}(T)=A_{2}\cdot T^{3}+B_{2}\cdot T^{2}+C_{2}\cdot T+D_{2}$$

Among them, *A*_2_, *B*_2_, and *C*_2_ are empirical parameters, with their specific coefficient values detailed in Table 5, *F*_21_(*T*) is the value of the resonance point of *S*_21_, *T* is the glucose solution temperature. This function clearly reflects the variation of *S*_21_ resonance points at different temperatures.

To better investigate the impact of temperature on experimental accuracy, a three-dimensional plot was generated using Functions (6) and (7). This plot visualizes the combined effect of glucose concentration and temperature on frequency, providing a clearer understanding of how temperature fluctuations influence the frequency response of the sensor. To enhance the precision of the results, temperature correction was applied. By accurately fitting Functions (6) and (7), a corrected mathematical model, represented by Function (8), was developed. This model effectively compensates for performance deviations caused by temperature changes, ensuring accurate measurements across different temperature conditions. Function (8) is as follows:

(8) $$F_{21}(T,c)=A_{3}+B_{3}\cdot T+C_{3}\cdot c+D_{3}\cdot T^{2}+E_{3}\cdot c^{2}+F_{3}\cdot T^{2}\cdot c^{2}$$

**Figure 12:**
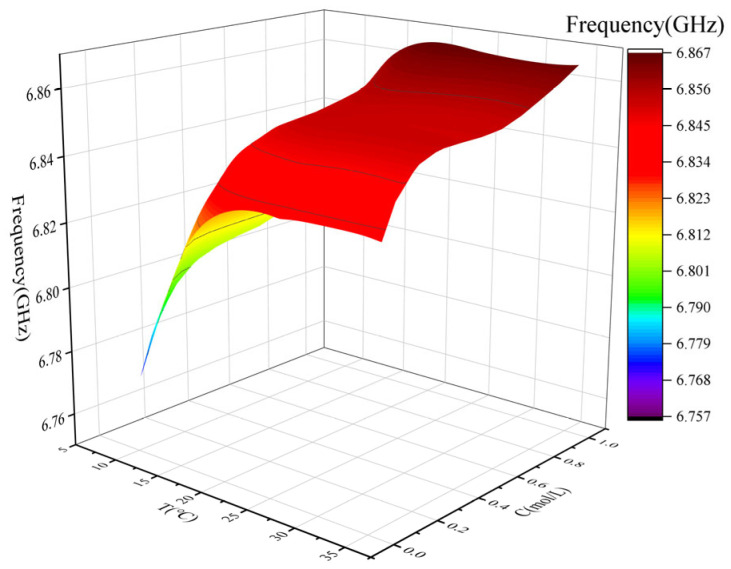
**Three-dimensional** surface plot of the lowest resonance point of the *S*_21_ parameter as a function of glucose concentration and temperature.

Among them, A_3_, B_3_, C_3_, D_3_, E_3_ and F_3_ are empirical parameters, with their specific coefficient values detailed in Table 6. Together they depict the coupled effect of temperature *T* and glucose concentration *c* on the lowest resonance frequency point of the *S*_21_ parameter. The relationship between the lowest resonance point of the *S*_21_ parameter as a function of glucose concentration and temperature is shown in Figure 12. Figure 12 illustrates the relationship between frequency, temperature, and concentration, revealing a complex nonlinear positive correlation among the three variables. The frequency reaches its minimum at approximately 9 °C and 0.05 mol/L and increases with either rising temperature or concentration. At a fixed concentration, frequency increases notably with temperature in the 5–20 °C range. Similarly, at a fixed temperature, frequency increases significantly with concentration in the 0.05–0.4 mol/L range. The surface plot also indicates an interaction effect: frequency is more sensitive to temperature variations at low concentrations, and more sensitive to concentration variations at low temperatures. When both temperature and concentration are high, the frequency approaches a saturation level above 6.85 GHz, and the surface becomes relatively flat, indicating a diminished response to further parameter increases. Overall, frequency exhibits an increasing trend with temperature and concentration, with a gradual saturation behavior in the high-value region.

**Figure 13:**
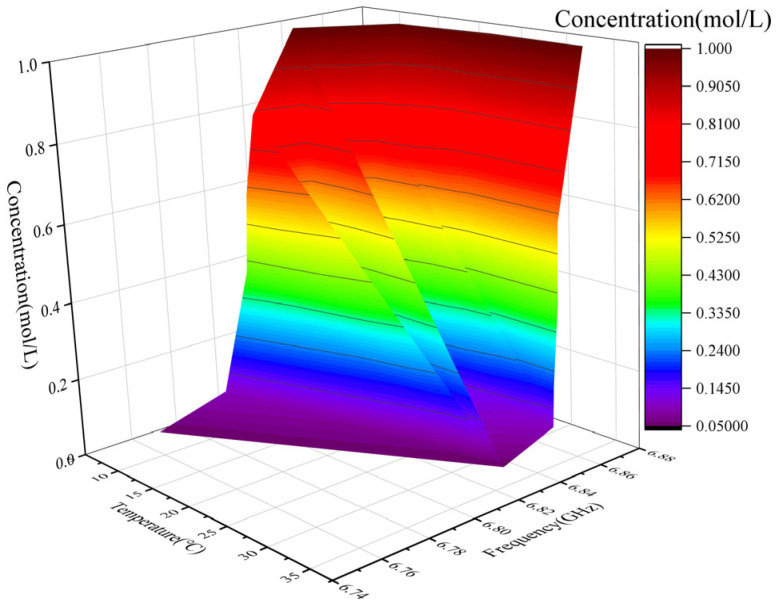
**Three-dimensional** surface plot of glucose concentration varying with temperature and frequency.

Figure 13 provides a comprehensive overview of temperature variations across different glucose concentrations and their impact on the *S*_21_ resonance point, emphasizing the critical role of temperature control in ensuring reliable glucose measurements. Figure 13 shows a three-dimensional surface diagram illustrating the relationship between solution concentration, temperature, and frequency. The color gradient in Figure 13 represents different concentration levels, with color changes indicating variations in concentration with temperature and frequency. The black area represents the lowest concentration approaching 0.05 mol/L, while the red area signifies the highest concentration reaching 1 mol/L. The surface morphology shown in Figure 13 exhibits complexity and volatility, indicating significant concentration changes with changes in temperature and frequency.

Variations in solution concentration affect frequency behavior. Figure 13 shows that the concentration exhibits a distinct step-like transition under different combinations of temperature and frequency, rapidly rising from approximately 0.05 mol/L to nearly 1.0 mol/L. At a fixed frequency, concentration typically undergoes a sharp increase near the critical temperature. Similarly, at a fixed temperature, there exists a critical frequency that causes the concentration to shift abruptly from a low to a high level. A strong coupling effect is observed between the critical conditions: at lower frequencies, achieving high concentration requires a higher temperature of around 25 to 30 °C, whereas at higher frequencies, a lower temperature around 15 to 20 °C is sufficient. This indicates that increasing frequency reduces the critical temperature required for high concentration, and vice versa. Therefore, temperature and frequency exhibit a clear synergistic or complementary effect in concentration regulation. Overall, the figure demonstrates that concentration is highly sensitive to both temperature and frequency, with a steep transition region that distinctly separates low and high concentration states.

## 4. Evaluation and Discussion

To ensure sensor performance reliability and robustness, a comprehensive statistical analysis is conducted. The performance index of the sensor is evaluated using the sensitivity calculation formula

(9) $$\;S=\frac{f_{1}-f_{0}}{(\varepsilon_{1}-\varepsilon_{0})\cdot f_{0}}\times 100\%$$

where *f*_1_ and *ε*_1_ represent the resonance frequency and dielectric constant of the medium, and *f*_0_ and *ε*_0_ denote the unloaded resonance frequency and dielectric constant. The calculated sensitivity is 0.54%. Sensitivity analysis shows that the output of the sensor changes proportionally with glucose concentration, but sensitivity decreases at higher temperatures. To further verify the performance of this sensor, a comparative analysis was conducted with other glucose solution sensors [51,56,57,58,59], as shown in Table 7, illustrating different performance characteristics. For instance, a CPW with an IDT sensor on an FR4 substrate (25.4 × 30 mm^2^) exhibits a sensitivity of 0.0153% and is independent of temperature. Similarly, the SIR sensor using the Rogers RT6002 substrate achieves a sensitivity of 0.44% and is also temperature-independent. The Minkowski-like sensor, utilizing the Rogers 5880 substrate, measures 27.5 × 60 mm^2^ with a sensitivity of 0.00035% and remains temperature-independent. Additionally, the CSRR sensor based on the Rogers RO3003 substrate (40 × 40 mm^2^) provides a sensitivity of 0.0016% and is also temperature-independent. Meanwhile, the MCSRR sensor on the FR4 substrate (35 × 25 mm^2^) has a sensitivity of 0.21%, maintaining temperature independence. In contrast, the microwave sensor on the FR4 substrate (20 × 30 mm^2^) stands out with a high sensitivity of 0.54%, albeit being temperature-dependent. Integrating a microfluidic temperature control method greatly enhances its practicality.

The microwave sensor excels in measuring glucose solutions with a sensitivity of 0.54%, demonstrating good responsiveness to low-concentration glucose solutions and the ability to detect subtle concentration changes. Introducing microfluidic temperature control methods significantly enhances its reliability and effectiveness in practical applications. These characteristics underscore the broad application prospects and significant practical value of the sensor in biomedical detection, the food industry, and other fields. Comparative analysis with other sensors further confirms its technological and performance advantages, demonstrating its great potential for future applications.

## 5. Conclusions

This paper designed a microfluidic microwave sensor on the twisted cross-shaped structure for glucose detection and analyzed the impact of temperature on its measurement accuracy. The operating frequency of this sensor is 6900 MHz, and its sensitivity is 0.54%. Meanwhile, in order to analyze the influence of temperature on the measurement accuracy, a constant-temperature water bath micro-circulation system was adopted. Under the isothermal conditions, an incremental increase of 0.2 mol/L in glucose concentration induces a resonance frequency shift of approximately 10–60 MHz in the scattering parameter. Conversely, under a fixed glucose concentration, a temperature rise of 10 °C results in a resonance frequency shift ranging from 10 to 130 MHz. Finally, the response relationship of this microwave sensor to different glucose concentrations and temperatures is very complex. Thus, a temperature compensation mathematical model is proposed, which effectively reduces the influence of temperature fluctuations on the performance of the sensor and thereby improves the measurement accuracy. This work provided a valuable insight for glucose monitoring and offered a basis for improving the accuracy and reliability of microwave sensors under different environmental conditions.

## Figures and Tables

**Table 1:** Parameters of different microwave sensors.

Geometry Type	Max Electric Field (V/m)	Average Sensitivity (%)
Cross-shaped	2.5 × 10^4^	0.39
Cross-CSRR	3.7 × 10^4^	0.48
Twisted-Cross	1.5 × 10^5^	0.54

**Table 2:** Parameters of the designed microwave sensor.

Parameter	Values (mm)
L	14
Z1	8
L2	0.5
L3	3
L4	2
L5	3
H	2.3
H1	2.5
R3	0.75
R4	1.1
W	0.35

**Table 3:** Microwave sensor equivalent circuit parameters.

Parameter	Value	Unit
L_L1_	0.1	nH
L_L2_	2.0	nH
L_L3_	0.1	nH
R	0.1	Ω
R1	0.1	Ω
R2	0.1	Ω
C1	5.2	pF
C2	0.3	pF
C3	0.5	pF
C4	1.4	pF
C5	5.15	pF

**Table 4:** Coefficients of Equation (6).

T (°C)	A_1_	B_1_	C_1_	D_1_	R^2^
9	1.692 × 10^7^	−1.419 × 10^7^	−7.934 × 10^5^	6.808 × 10^9^	0.929
15	7.092 × 10^6^	−6.362 × 10^6^	5.217 × 10^6^	6.843 × 10^9^	0.967
25	9.542 × 10^6^	−9.021 × 10^6^	−6.234 × 10^5^	6.854 × 10^9^	0.925
35	3.031 × 10^6^	−1.011 × 10^6^	1.294 × 10^6^	6.856 × 10^9^	0.995

**Table 5:** Coefficients of Equation (7).

C (mol/L)	A_2_	B_2_	C_2_	D_2_	R^2^
0.05	4.094 × 10^7^	−2.839 × 10^7^	−1.031 × 10^7^	6.829 × 10^9^	0.99
0.1	2.745 × 10^6^	−1.715 × 10^7^	2.613 × 10^7^	6.841 × 10^9^	0.99
0.2	1.961 × 10^7^	−2.429 × 10^7^	3.938 × 10^7^	6.849 × 10^9^	0.99
0.4	1.426 × 10^7^	−1.955 × 10^7^	7.434 × 10^6^	6.850 × 10^9^	0.99
0.6	2.104 × 10^7^	−2.264 × 10^7^	−1.612 × 10^6^	6.854 × 10^9^	0.99
0.8	1.570 × 10^7^	−2.215 × 10^7^	4.386 × 10^6^	6.858 × 10^9^	0.99
1.0	1.488 × 10^7^	−1.715 × 10^7^	−1.424 × 10^6^	6.866 × 10^9^	0.99

**Table 6:** Coefficients of Equation (8).

A_3_ × 10^9^	B_3_ × 10^9^	C_3_ × 10^9^	D_3_ × 10^9^	E_3_ × 10^9^	F_3_ × 10^9^	R^2^
6.7793	0.0042	0.021	0.0026	−0.2	0.1422	0.98

**Table 7:** Comparison of various microwave sensors.

Ref.	Sens. Method	Substrate	Size (mm^2^)	Sensitivity (%)	Measurement Error	Temperature Control or Not
[56]	CPW-IDT	FR4	25.4 × 30	0.0153	-	NO
[51]	SIR	Ro6002	-	0.44	±1.3–7.1%	NO
[57]	Mink-like	Ro5880	27.5 × 60	0.00035	-	NO
[58]	CSRR	Ro3003	40 × 40	0.0016	±1.8%	NO
[59]	MCSRR	FR4	35 × 25	0.21	±5%	NO
This work	Twisted Cross-Shaped	F4B	20 × 30	0.54	±1.1–4.3%	YES

## Data Availability

Data are available upon request.
